# A neural network with a human learning paradigm for breast fibroadenoma segmentation in sonography

**DOI:** 10.1186/s12938-024-01198-z

**Published:** 2024-01-14

**Authors:** Yongxin Guo, Maoshan Chen, Lei Yang, Heng Yin, Hongwei Yang, Yufeng Zhou

**Affiliations:** 1 State Key Laboratory of Ultrasound in Medicine and Engineering, College of Biomedical Engineering, Chongqing Medical University, 1 Medical College Road, Chongqing, 400016 China; 2 Chongqing Key Laboratory of Biomedical Engineering, Chongqing Medical University, Chongqing, 400016 China; 3 Department of Breast and Thyroid Surgery, Suining Central Hospital, Suining, 629000 China; 4 National Medical Products Administration (NMPA) Key Laboratory for Quality Evaluation of Ultrasonic Surgical Equipment, 507 Gaoxin Ave., Donghu New Technology Development Zone, Wuhan, 430075 Hubei China

**Keywords:** Breast fibroadenomas, Sonography, Segmentation, Human learning paradigm, Deep learning, CNN–transformer hybrid model

## Abstract

**Background:**

Breast fibroadenoma poses a significant health concern, particularly for young women. Computer-aided diagnosis has emerged as an effective and efficient method for the early and accurate detection of various solid tumors. Automatic segmentation of the breast fibroadenoma is important and potentially reduces unnecessary biopsies, but challenging due to the low image quality and presence of various artifacts in sonography.

**Methods:**

Human learning involves modularizing complete information and then integrating it through dense contextual connections in an intuitive and efficient way. Here, a human learning paradigm was introduced to guide the neural network by using two consecutive phases: the feature fragmentation stage and the information aggregation stage. To optimize this paradigm, three fragmentation attention mechanisms and information aggregation mechanisms were adapted according to the characteristics of sonography. The evaluation was conducted using a local dataset comprising 600 breast ultrasound images from 30 patients at Suining Central Hospital in China. Additionally, a public dataset consisting of 246 breast ultrasound images from Dataset_BUSI and DatasetB was used to further validate the robustness of the proposed network. Segmentation performance and inference speed were assessed by Dice similarity coefficient (DSC), Hausdorff distance (HD), and training time and then compared with those of the baseline model (TransUNet) and other state-of-the-art methods.

**Results:**

Most models guided by the human learning paradigm demonstrated improved segmentation on the local dataset with the best one (incorporating C3ECA and LogSparse Attention modules) outperforming the baseline model by 0.76% in DSC and 3.14 mm in HD and reducing the training time by 31.25%. Its robustness and efficiency on the public dataset are also confirmed, surpassing TransUNet by 0.42% in DSC and 5.13 mm in HD.

**Conclusions:**

Our proposed human learning paradigm has demonstrated the superiority and efficiency of ultrasound breast fibroadenoma segmentation across both public and local datasets. This intuitive and efficient learning paradigm as the core of neural networks holds immense potential in medical image processing.

## Background

Breast fibroadenomas are common, benign fibro-epithelial lesions [1], which are frequently encountered in adolescent girls and young women [2–4]. About 10% of women have such symptoms in their lifetime, accounting for 67–94% of all breast biopsies in women under the age of 20 years [5, 6]. Although mammography is the gold standard in the detection and evaluation of masses in the breast, sonography has become an indispensable imaging modality because of its technical advantages of non-ionization, low cost, mobility, and real-time diagnosis [7]. However, the low spatial resolution and image quality of sonography make it hard to extract tissue morphological features accurately and reliably. Breast sonography is thus highly operator-dependent and has a high inter-observer variation rate [8]. To reduce the burden for the radiologist in reviewing hundreds of clinical images and improve the accuracy of diagnosis, computer-assisted image segmentation becomes valuable.

Deep learning approaches are increasingly being used for medical image segmentation and quantitative information regarding the morphology and textural features of lesions [9]. Several neural network architectures developed from convolutional neural networks (CNNs) have shown satisfactory segmentation performance. However, breast ultrasound (BUS) image segmentation is still challenging due to high speckle noise, a low contrast, blurry boundaries, and intensity inhomogeneity in sonography [10]. Therefore, precisely segmenting breast fibroadenoma in sonography requires extensive investigation, and deep learning models with the capability of processing more complex textures, focusing on the most important features, increasing robustness, and having noise immunity are preferred.

**Fig. 1:**
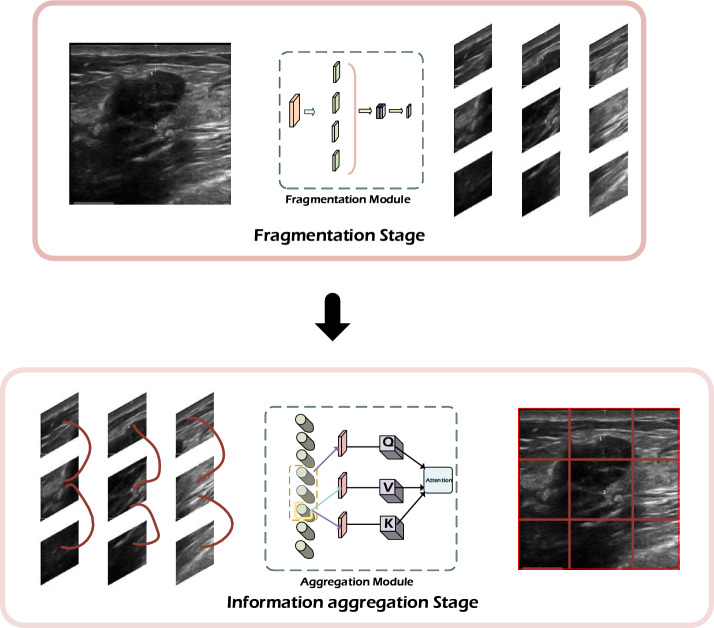
A workflow that mimics the human learning paradigm, and the red curves at the information aggregation stage are the relationships between each slice

There have also been various attempts to incorporate domain knowledge into neural networks in medical image analysis, such as diagnosis, detection, and segmentation [11]. Some notable approaches include transfer learning, teacher–student course learning, and combined attention maps. Transfer learning involves leveraging knowledge from natural images to guide medical image analysis. By using a pre-trained network as a fixed feature extractor, knowledge can be transferred between image domains. Although these approaches are promising, simulating the natural learning process observed in humans may be another strategy in deep learning. Humans typically break down complex information into smaller, manageable chunks during learning and then integrate them according to their inherent relationships, which facilitates the learning of large-scale information more effectively. Thus, we propose a human instinct learning paradigm that involves feature fragmentation and information aggregation as a guide for neural network learning to enhance the segmentation performance of breast fibroadenoma in sonography. The workflow of our proposed learning paradigm is shown in Fig. 1.

In this study, we propose an efficient paradigm that emulates the intuitive human learning mechanisms within an artificial neural network to guide the segmentation of breast fibroadenomas in sonography. Feature fragmentation attention modules (Focus, BottleneckCSP, and C3ECA) and information aggregation modules (LogSparse Attention, C3CBAM, and ProbSparse Attention) were selected and specifically tailored to the characteristics of ultrasound images. A dataset of breast ultrasound images of Asian women at Suining Central Hospital, China was constructed, and then the validation and performance of our proposed lightweight model were tested and evaluated on both local and public datasets. Furthermore, our approach was compared with other state-of-the-art (SOTA) methods to confirm its superior segmentation performance.

### Related works

#### Deep learning-based network

U-Net is one of the most popular and outstanding networks [12]. However, it cannot learn global and long-range semantic information interaction well due to the locality of the convolution operation. The self-attention mechanism and sequence-to-sequence design of transformers work effectively in the global extraction of contextual information, being extensively successful in natural language processing (NLP) [13]. Cao et al. proposed a pure transformer-based U-shaped encoder–decoder model for medical image segmentation [14]. Furthermore, the CNN–transformer hybrid network demonstrates great segmentation performance. Schlemper et al. integrated the attention gate module into the encoder–decoder design of the U-shaped architecture [15]. In addition, TransUNet combines the best features of CNN in processing high-dimensional data with the transformer’s ability to capture location and contextual information [16]. This model can hold more than 100B trainable parameters, but at a significantly increased computational burden [17, 18].

#### Breast ultrasound image segmentation

Deep CNNs have been applied to lesion segmentation in BUS images [19, 20]. A fuzzy CNN model incorporating data enhancement as well as fine-tuning post-processing is proposed by Huang et al. for BUS image segmentation [21]. Xue et al. designed a neural network with a global guidance module as well as a breast lesion boundary detection module, whose outcomes are further optimized by pre-defined regularization conditions to improve the segmentation accuracy [22]. Similarly, Lei et al. introduced boundary regularization into deep convolutional encoder–decoder networks to reduce the influences of noise and other factors on BUS images [23]. Abdelali et al. presented an automated CAD system for breast cancer detection and classification in mammography utilizing multiple instance learning (MIL) algorithms in decision-making [24]. Furthermore, suspicious regions were assessed in screening mammography at an impressive sensitivity of 98.60% using a modified K-means algorithm for region segmentation and bi-dimensional empirical mode decomposition (BEMD) for feature extraction [25].

#### Attention mechanisms

Attention mechanisms that emulate human perception have recently been introduced to neural networks [26] to select the more critical features from an extremely large amount of information by classifying feature maps channel-by-channel [27]. There are two main classes in the practice. The first class chunks the feature information channel-by-channel and then reassembles it. The Focus module, derived from Yolo v5 [28], reconstructs low-resolution images by selecting pixels from the original one and stacking adjacent pixels using dilated convolution [29]. This approach is also adopted by the Concentration-Comprehensive Convolution (C3) block [30], which enhances the network depth while reducing the computational complexity. For further optimization, a Bottleneck module replaces a single large-sized convolution with multiple small-sized convolutions [31]. Combining it with the cross-stage partial network (CSPNet) gives rise to the BottleneckCSP module to improve memory consumption and learning efficiency [32]. These findings suggest that piecewise learning of feature information can enhance model efficiency.

The second class of attention mechanisms aims to enhance the extraction of semantic information by encoding the feature map’s location information for contextual perception. The Efficient Channel Attention (ECA) module employs a local cross-channel interaction strategy to facilitate information interaction [33]. The LogSparse Transformer addresses the prediction accuracy of time series with fine-grained and strong long-term dependencies within memory constraints [34]. The Convolutional Block Attention Module (CBAM) provides attention weights in both channel and spatial dimensions, aiding in the extraction of effective target features [35]. Additionally, a new formulation of attention through the kernel lens provides a deeper understanding of attention components and enhances the dynamics and utilization of the transformer's multi-headed self-attention mechanism [31]. Despite the promising performance of these attention modules, their application in medical imaging is still limited.

#### Knowledge-based methods

Maicas et al. proposed a teacher–student curriculum learning strategy that mimics more challenging tasks, such as breast image classification for dynamic contrast-enhanced magnetic resonance imaging (DCE-MRI) [36]. Emulating this process in the neural network training improved the classification performance by 5.88% compared to the baseline, DenseNet. An attention-based CNN for glaucoma detection, AG-CNN, combined attention maps during the supervised training process and simulates the physicians’ focus on regions of interest [37]. This method allows the network to learn from the attention patterns of medical professionals and enhance its ability to identify the most relevant features. Hsu et al. incorporated the existing BI-RADS (Breast Imaging Reporting and Data System) score [38] as the knowledge to guide the neural network in learning the texture and intensity features of BUS images [39]. A learning paradigm that goes beyond the confines of medical knowledge to guide neural networks in medical image processing and uses human learning paradigm may provide a more versatility.

## Results

We utilized both local and public datasets to validate the effectiveness of our proposed learning paradigm and conducted ablation experiments to explore its interpretability.

### Performance assessment

Table 1 lists the outcomes of the proposed CNN–transformer hybrid network using different fragmentation and aggregation modules on the local dataset. Appropriate network architectures show improvements in the evaluation metrics as well as a reduction in the training time. The most significant results came from combining the C3ECA and LogSparse Attention modules, 0.876 in DSC and 5.82 mm in HD, respectively. Compared with the baseline model (TransUNet), the corresponding improvements are 0.76% in DSC and 3.51% in HD, respectively. The computation time is reduced by 1.25 h. The impact of incorporating different fragmentation modules on segmentation performance and training time is also illustrated. Notably, the model utilizing C3ECA as the fragmentation module achieves the overall shortest training time within the range of 2.25–2.75 h.

**Table 1:** Comparison of the segmentation performances on the local dataset using the CNN–transformer hybrid network containing different modules with the best results in bold

Methods		DSC ↑	HD (mm) ↓	Time (h)↓
Fragmentation module	Aggregation module			
NA	NA	0.869240	8.963769	4
Focus	LogSparse	0.870811	8.026729	4
	C3CBAM	0.867615	7.836283	3.75
	ProbSparse	0.869836	8.212088	3
BottleneckCSP	LogSparse	0.874429	7.846115	3.25
	C3CBAM	0.855873	10.848110	**2.25**
	ProbSparse	0.854521	9.704177	3.25
C3ECA	LogSparse	**0.875815**	**5.820322**	2.75
	C3CBAM	0.870480	8.873851	**2.25**
	ProbSparse	0.863999	9.969885	2.75

**Fig. 2:**
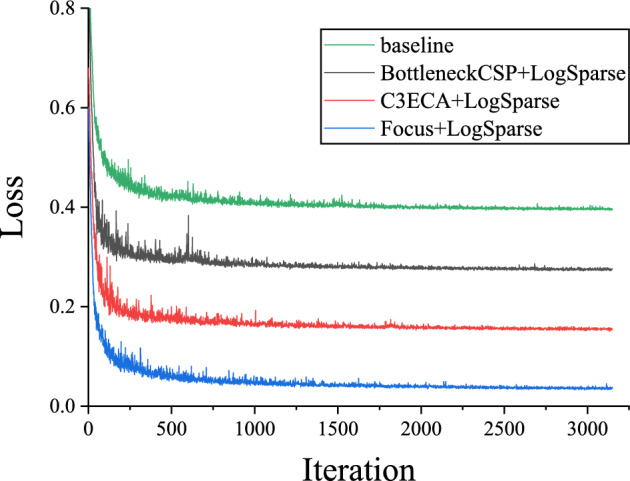
Comparison of loss curves produced by the baseline model (TransUNet) and CNN–transformer hybrid network containing the modules of BottleneckCSP + LogSparse, C3ECA + LogSparse, and Foucs + LogSparse with uniform coordinate offsets of 0.13

To assess whether the human learning paradigm introduces overfitting issues during training, we analyzed the training losses of the model incorporating the LogSparse Attention module (see Fig. 2). The results clearly indicate that the inclusion of C3ECA module achieves the fastest fitting and convergence and the lowest loss with gradually diminishing loss jitters even after only 250 iterations. In contrast, the model incorporating BottleneckCSP exhibits higher loss jitters around the 700th iteration (i.e., up to 0.128). Overall, it shows that the neural network guided by the human learning paradigm is capable of finding the local optimal solutions more efficiently at faster convergence in the training process.

### Visualizations

**Fig. 3:**
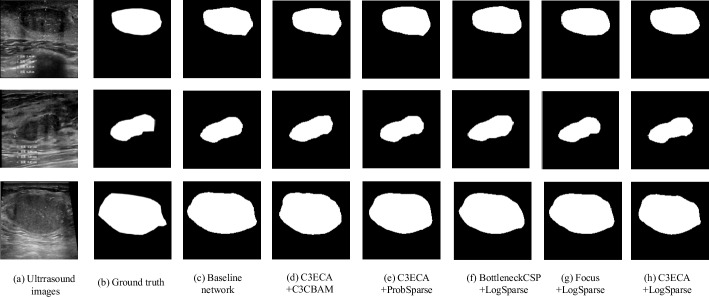
Comparison of **a** the breast ultrasonic image, **b** the ground truth masks, and the predicted masks using **c** baseline, **d** C3ECA and C3CBAM, **e** C3ECA and probSparse, **f** BottleneckCSP and LogSparse, **g** Focus and LogSparse, and **h** C3ECA and LogSparse for three representative cases of breast fibroadenoma I (top row): a conventional fibroadenoma; II (middle row): a fibroadenoma with an overall longer cross-section; III (bottom row): a fibroadenoma with an overall larger area

**Fig. 4:**
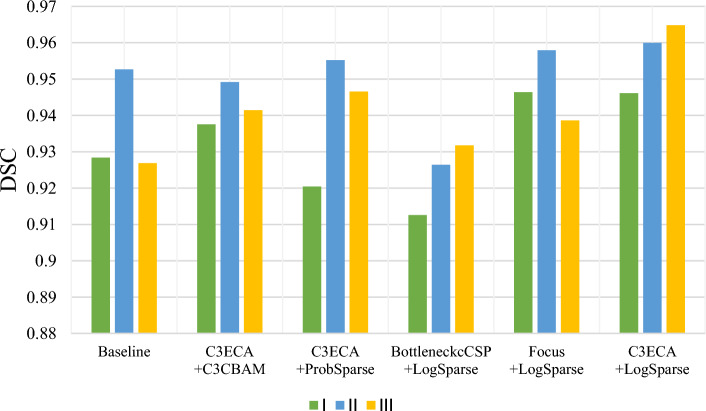
Quantitative comparison of the segmentation DSC metrics of the breast fibroadenomas using different networks

A visual comparison of the segmentation results for three representative cases: Case I, a conventional fibroadenoma; Case II, a fibroadenoma with an overall longer cross-section; and Case III, a fibroadenoma with an overall larger area and the corresponding DSC metrics are shown in Figs. 3 and 4, respectively. Our findings reveal that the LogSparse-related models demonstrate successful segmentation of all breast fibroadenomas with various pathological characteristics, resulting in segmentation contours that closely match the ground truth. However, the C3CBAM- and ProbSparse-related models exhibit some misclassifications, particularly in Case I with incorrect expansions in the segmented lesion boundaries. In Case II, the network model containing Focus and LogSparse modules performs the best. However, in Case III, the model combining C3ECA and LogSparse modules excels in predicting the conspicuous lower right part of the fibroadenoma. These results suggest that the model incorporating the human learning paradigm, especially the combined C3ECA and LogSparse Attention modules, demonstrates improvements over the baseline model.

### Comparison to state-of-the-art methods

**Fig. 5:**
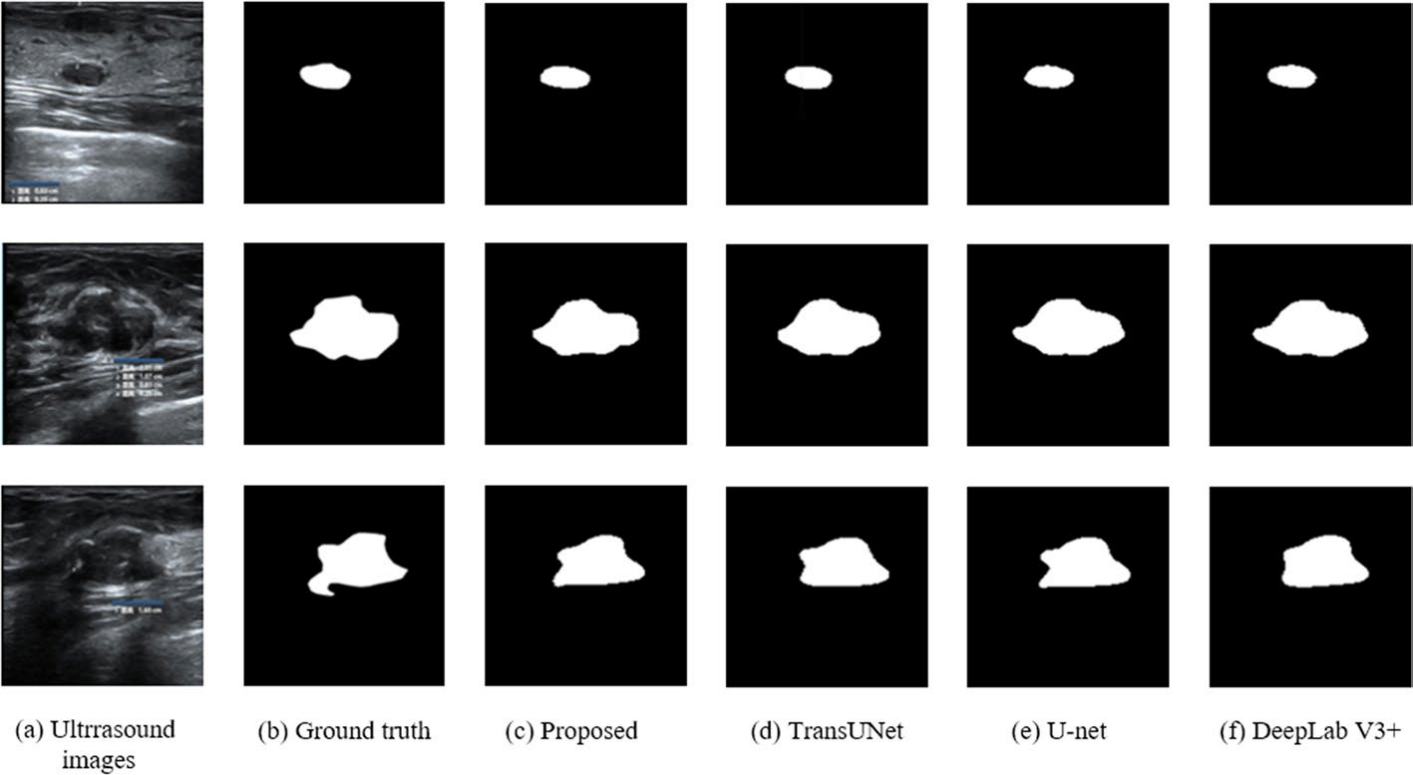
Visual comparison of **a** three representative ultrasound breast fibroadenoma images from the local dataset, **b** ground truth, and segmentation results using **c** our proposed model and state-of-the-art methods of **d** TransUNet, **e** U-net, and **f** DeepLab V3 +

To further evaluate the performance of our work, the best-performing model (the combined C3ECA and LogSparse Attention modules) was compared with the SOTA models using a local dataset. Table 2 and Fig. 5 show that our proposed network enhances DSC and HD metrics by 6.1% and 4.3 mm and 3.82% and 3.46 mm, respectively, as compared to the U-net and DeepLab V3 + , which may be due to the emphasis on semantic information interaction. In comparison to U-net which has the least training time among all tested SOTA models, our approach reduces the training time by another 0.25 h.

**Table 2:** Comparison of the segmentation performances of three SOTA networks and ours on the local ultrasound breast images dataset with the best results in bold

Network	DSC ↑	HD (mm) ↓	Time (h)↓
TransUNet	0.869240	8.963769	4
U-Net [12]	0.825394	10.121881	3
DeepLab V3 + [40]	0.843596	9.281189	3.25
Proposed	**0.875815**	**5.820322**	**2.75**

### Robustness on the public dataset

**Fig. 6:**
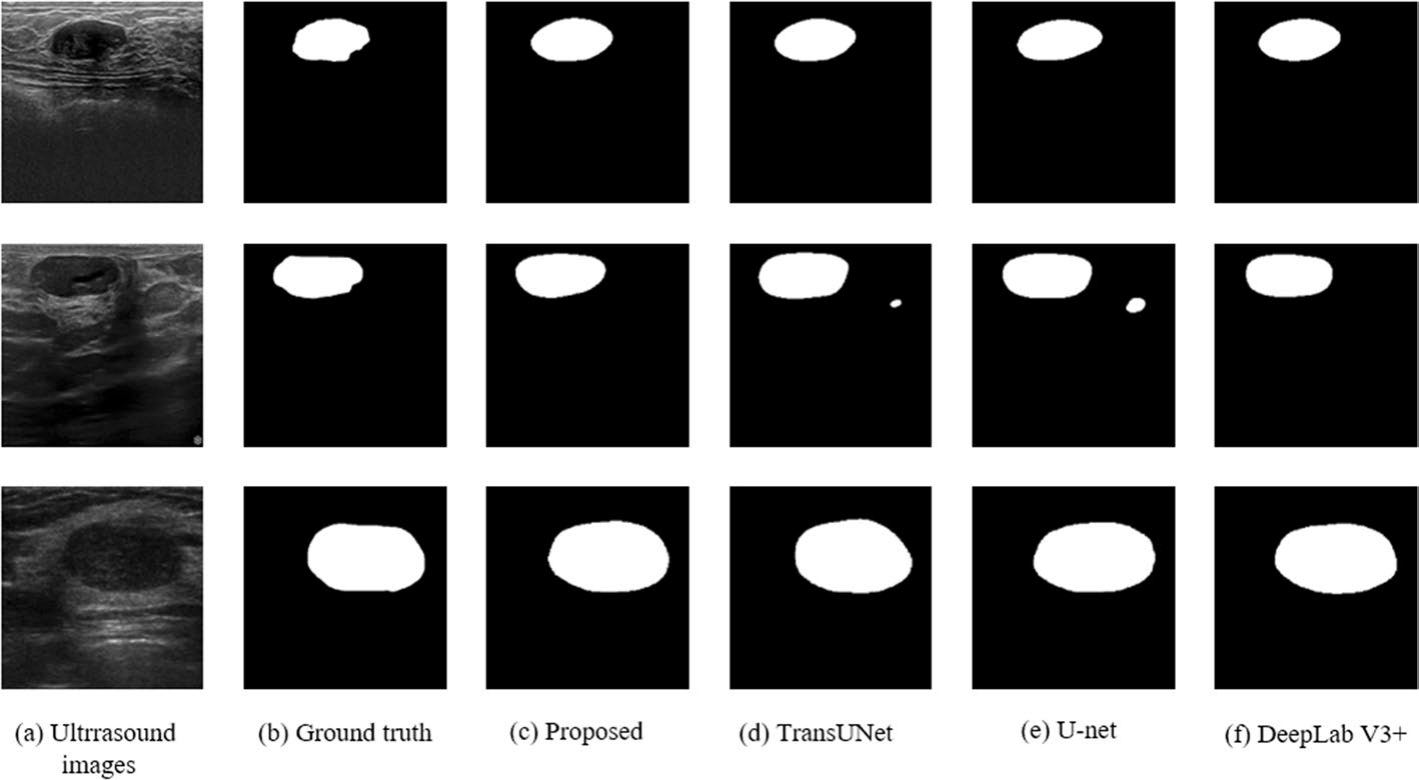
Visual comparison of **a** three representative ultrasound breast fibroadenoma images from the public dataset, **b** ground truth, and segmentation results using **c** our proposed model and state-of-the-art methods of **d** TransUNet, **e** U-net, and **f** DeepLab V3 +

To further validate the robustness of our learning paradigm, we conducted tests on the publicly available dataset (Dataset_BUSI and DatasetB) to compare with the SOTA model. Table 3 and Fig. 6 show that our network is also applicable to the public dataset quite well, outperforming TransUNet by 0.42% in DSC and 5.13 mm in HD and DeepLab V3 + by 1.43 mm in HD, respectively. However, the training time optimization embodied in our lightweight model for the local dataset becomes less pronounced here, which may be due to the smaller sample size (500 vs. 207). Because of the more complicated structure of artificial neural networks than these traditional linear ones [41], the training time is not proportional to the sample size.

**Table 3:** Comparison of the segmentation performances using three SOTA networks and ours on the public ultrasound breast dataset with the best results in bold

Network	DSC ↑	HD (mm) ↓	Time (hr)↓
TransUNet	0.849274	38.664333	2.5
U-Net	0.832526	37.099123	2.25
DeepLab V3 +	0.852061	35.231811	**2**
Proposed	**0.852804**	**33.796840**	2.25

### Ablation study

To investigate the effect of the fragmentation module (C3ECA) and aggregation module (LogSparse) on the neural network in improving breast fibroadenoma segmentation, these two modules were integrated into the baseline model individually. Each hyperparameter in the experiment and clinical dataset was maintained consistently to ensure the fairness. The network containing the fragmentation module exhibits a significant decrease in training time but a minor improvement in DSC, while the inclusion of the aggregation module improves DSC by 0.56% and HD by 0.17 mm (see Table 4). More importantly, the performance of the combined fragmentation and aggregation modules is much better than that of the individuals (i.e., by 3.38 mm and 2.97 mm in HD, respectively). Therefore, such a combination is synergistic in the learning paradigm.

**Table 4:** Ablation analysis of different components in the proposed network with the best results in bold

Network	DSC ↑	HD (mm) ↓	Time (h)↓
baseline	0.869240	8.963769	4
baseline + C3ECA	0.873358	9.295890	2.5
baseline + LogSparse	0.874087	8.790277	4.75
baseline + C3ECA + LogSparse	**0.875815**	**5.820322**	**2.75**

## Discussion

In this study, we present a novel approach to enhance the segmentation of breast fibroadenomas in sonography through the utilization of an artificial neural network with a human learning paradigm. Our method is inspired by the learning mechanisms of the human brain, and the new paradigm combines feature fragmentation modules (Focus, BottleneckCSP, and C3ECA) and information aggregation modules (C3CBAM, LogSparse Attention, and ProbSparse Attention) to effectively guide the neural network's learning process. To validate its effectiveness, we conducted a comprehensive set of experiments using both local and public datasets. The quantitative evaluation demonstrates the superiority of our model over state-of-the-art models in terms of Dice similarity coefficient (DSC) and Hausdorff distance (HD) metrics, while also significantly reducing training time. Remarkably, the network employing C3ECA and LogSparse Attention mechanisms showcased the most exceptional performance on both datasets, improving DSC and HD by 0.76% and 3.51% on the local dataset and by 0.42% and 12.59% on the public dataset compared to the TransUNet model, respectively. Altogether, our study introduces an artificial neural network framework augmented by a human-inspired learning paradigm that effectively enhances the segmentation of breast fibroadenomas.

**Fig. 7:**
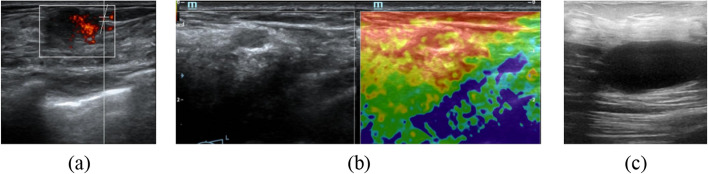
Representative ultrasound image with **a** the arterial blood flow signal, **b** B-mode sonography on the left and elastography on the right in the local dataset, and **c** B-mode sonography in the public dataset_BUSI

Considering the variations in breast densities and anatomical features among populations, particularly between Asian and European-American women, our study holds clinical relevance for early breast fibroadenoma diagnosis. It is also important to note that the image quality of sonography varies greatly due to the operating equipment. In our data collection, ultrasound images were acquired from local women in Suining, a representative small to medium-sized city in China, using the sonographic equipment of the Mindray DC-80S. Their qualities are found poorer compared to those in the public datasets that consist of the BUS images in Europe and North America (see Fig. 7). However, the consistently outstanding performance (i.e., robustness and reliability) of our models across diverse datasets showcases their potential and value for clinical applications.

The future of computer vision research is likely to focus on targeted and guided feature learning. Self-attention mechanisms, derived from transformers, are competitive, and many variants have been developed. While attention mechanisms improve contextual information extraction, their performance in sonography, which contains uniformly distributed complex patterns, is unsatisfactory. Filipczuk et al. used a k-means based hybrid method for beast fibroadenomas segmentation, but at an average classification accuracy of only 77.20% [42]. In our work, inspiration from the human’s learning pattern led to devising a fragmentation–aggregation learning paradigm and then incorporating it with a feature segmentation method. This method involves partitioning and allocating feature maps to distinct channels, culminating in the comprehensive acquisition of information and the emulation of the human’s learning trajectory, and progressing from surface-level to in-depth understanding and from local to global comprehension. Rather than indiscriminately adding modules to the neural network, focusing on specific scenarios to optimize performance seems more effective. Here, this learning paradigm was adapted into a mechanism encompassing both fragmentation focus and information aggregation for improved segmentation and streamlined architectures. Ablation experiments have validated its synergistic benefits.

This study has some limitations. Firstly, the size of our dataset is modest compared to other publicly available medical image datasets (i.e., MRI and CT). However, we plan to continuously collect more breast ultrasound images (i.e., 300 ones over the next four months), which will significantly enhance the dataset's size and diversity. Although a promising DSC of 0.875815 was achieved here, there is still ample room for further investigation and improvement. Future studies should focus on exploring novel techniques and continuously refining the learning paradigm to enhance the accuracy and effectiveness of sonography segmentation. We will combine the online transfer learning strategy with the CNN–transformer hybrid network model [43], apply the feature-based transfer learning method [44], and migrate the SOTA methods of medical image segmentation, such as fuzzy c-means (FCM), Gaussian mixture model (GMM) [45], and the topology-preserving approach [46]. Furthermore, specific segmentation requirements will be explored. Ding et al. suggested that the segmentation of the brachial plexus could be transformed into a segmentation of the nerve as well as the surrounding tissues [47]. Therefore, blood flow and tissue elasticity signals may assist in segmenting the breast fibroadenomas in sonography (see Fig. 7). Finally, image segmentation will be evaluated in line with clinical practice and the physician’s intuitive judgment. DSC and HD illustrate only the geometric differences without considering the clinical implications. The smooth lesion boundaries and their tendency toward concavity can influence the physician's assessment of the tumor's benignity and malignancy in clinical diagnosis. Under- or over-contouring tumors with similar DSC and HD measures may lead to significantly different diagnosis results. A medical similarity index (MSI) that involves a user-defined medical consideration function (MCF) derived from an asymmetric Gaussian function will be used for evaluating the segmentation accuracy since the MCF shape shows the anatomical position and characteristics of a particular tissue, organ, or tumor type [48]. A subjective evaluation will also be applied. Although human evaluation is very cumbersome and time-consuming, it provides more clinical insights into the tumors. And the acceptance by experienced radiologists of the image segmentation results is critical in the clinical applications. Accurate medical image segmentation is a complex and challenging task due to significant variations in image quality, artifacts, and anatomical structures among patients. Thus, further investigation is required for the technical development and its translation to the clinics.

## Conclusion

Although sonography is preferred in the diagnosis of breast masses, segmentation of the tumors in sonography is unsatisfactory because of the inherent limitations of this imaging modality, low image quality and the presence of artifacts (i.e., speckles and scattering). Utilizing prior knowledge to guide neural network learning can lead to improved performance in specified medical image segmentation tasks. In this paper, we applied a paradigm inspired by human learning patterns to an artificial neural network for the segmentation of breast fibroadenomas in sonography. Our research findings indicate that aggregating high-dimensional information into cohesive modules can enhance the model's information perception ability while reducing training costs. By introducing three fragmentation attention modules and three information aggregation modules, we successfully implemented this learning paradigm and guided the neural network's learning process. Improvements in performance metrics across various network structures are found in comparison to the baseline network. Among all combinations, the C3ECA and LogSparse Attention modules showed the best overall segmentation performance in DSC, HD, and training time. Additionally, our approach demonstrated robust advantages over other state-of-the-art methods on both local and public breast ultrasound image datasets. This study underscores the immense potential of a modular learning paradigm inspired by the human brain within the realm of image processing. Although our findings are promising, there exists ample room for further exploration and refinement of this approach. Additional images will be incorporated into the dataset for a more comprehensive evaluation of our method's capabilities. Ultrasonic elastography may be utilized for capturing intricate mechanical features of breast masses. This strategic augmentation holds the promise of achieving more precise lesion segmentation.

## Methods

### Image data set

The dataset consists of clinical breast ultrasound images, including both our local dataset and a supplementary one using publicly available sources. The local dataset was collected retrospectively at Suining Central Hospital in Sichuan, China. The images were acquired from patients who underwent breast ultrasound examinations between January and July 2022. During the image acquisition, a sonographer systematically scanned the outer lower, outer upper, inner upper, and inner lower quadrants of the breast in a clockwise manner. Suspected lesions were analyzed, and the periareolar area and armpit were examined to determine the location and size of the lesions at both sagittal and cross-sectional viewing angles. Multiple ultrasound images were acquired for each fibroadenoma case according to the standard protocols. The image acquisition was performed by professional sonographers using a DC-80S system (Mindray Medical, Shenzhen, China). Overall, our local dataset comprises 600 breast ultrasound images obtained from 30 patients. Table 5 summarizes the basic information of all patients included in our local dataset. To supplement our local dataset and further validate the validity and robustness of our model, a professional sonographer selected benign fibroadenoma images from publicly available datasets, Dataset_BUSI [37] and DatasetB [38], and excluded those with ambiguous performance. Finally, 207 images from Dataset_BUSI and 39 images from DatasetB with their corresponding labels were merged as a public dataset. To ensure proper segmentation evaluation, all images on both local and public datasets are randomly divided into training and test sets in a 5:1 ratio in our experiments.

**Table 5:** Basic information of the patients and their breast fibroadenomas in the local dataset

Number of patients		30
Age		27–59, 41.978 $\pm$ 9.029
Tumor distribution	Single	21
	Multiple	9

### Network architecture

**Fig. 8:**
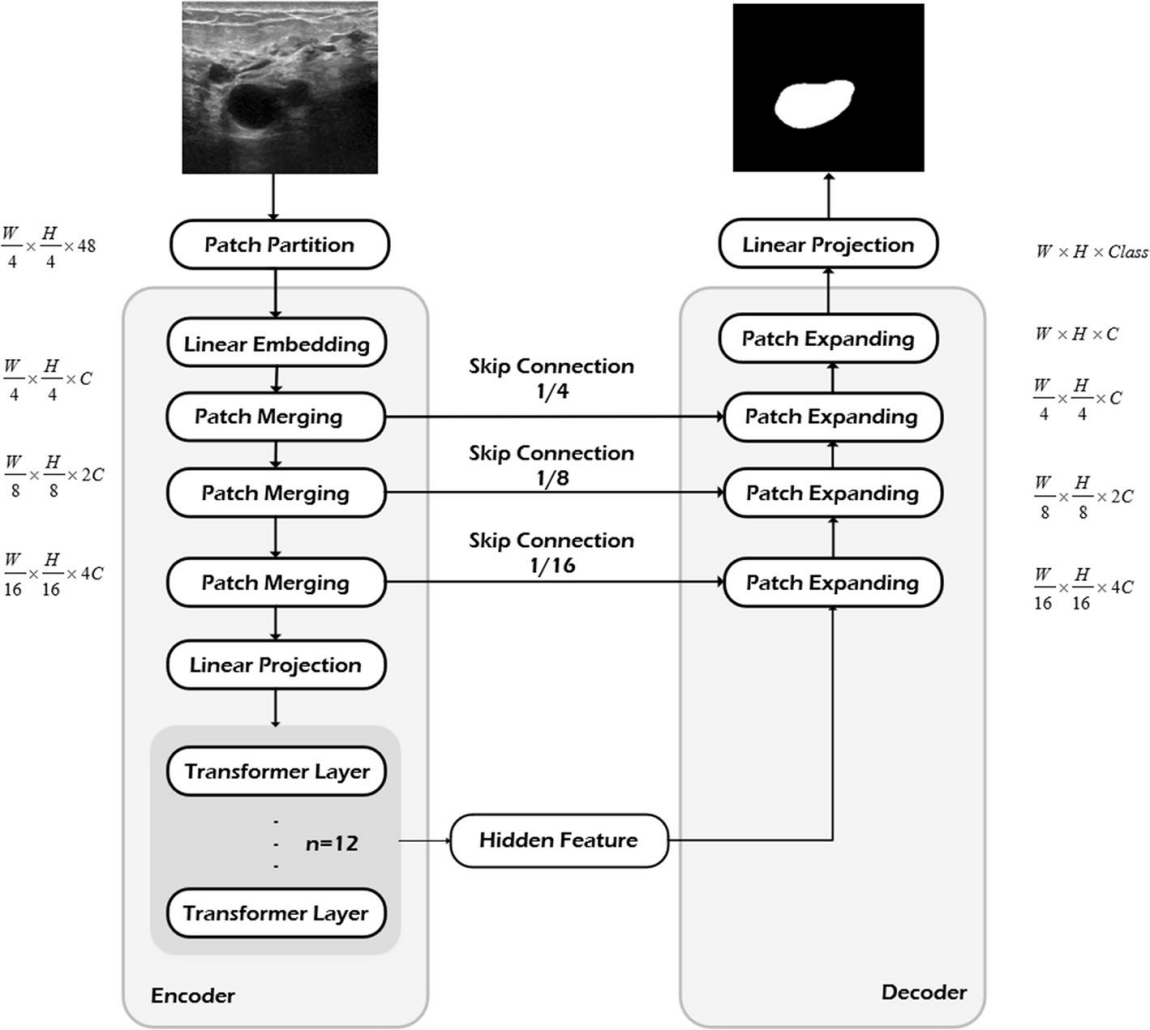
Architecture of the TransUNet used for segmenting the breast fibroadenomas in sonography

**Fig. 9:**
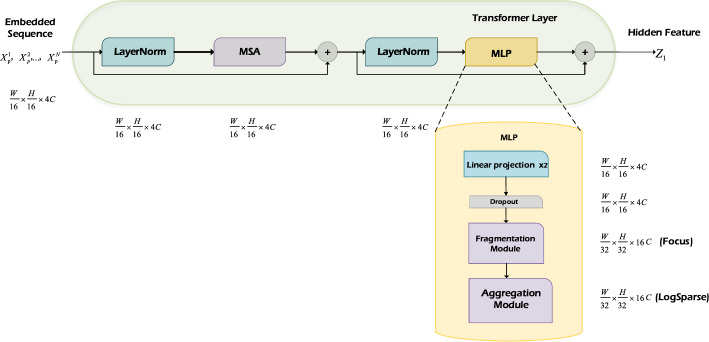
The structure of the transformer layer and multi-layer perceptron (MLP)

To implement the segmentation of breast fibroadenomas in sonography, we designed a model based on the framework of TransUNet, utilizing an encoder–decoder architecture (see Fig. 8). The process begins by reshaping the input image into a series of 2D patches using the patch partition parts. These patches are then vectorized and mapped to an embedding space using a trainable linear projection while preserving the positional information. Patch merging and expanding are responsible for downsampling and upsampling tasks, respectively. In Fig. 8, the dimensional changes of the input feature map are annotated. During each downsampling, the width and height are halved, while the number of channels is doubled (from $\frac{W}{4}\, \times \,\frac{H}{4}\, \times \,C$ to $\frac{W}{8}\, \times \,\frac{H}{8}\, \times \,C$). Conversely, in the upsampling process, the dimensional changes occur in the opposite direction. Typically, feature maps do not change in dimensions after being processed by the transformer layer. However, incorporation of the human learning paradigm within the transformer layer introduces dimension transformations based on the operations of different fragmentation and aggregation modules. Figure 9 provides an explanation of the feature map's transformations. The transformed patches are subsequently passed through the transformer layers, where the hidden layer features are extracted using the multi-head self-attention mechanism (MSA) and multi-layer perceptron (MLP).

At the decoder block, the image undergoes multiple layers of upsampling, and feature fusion is performed to generate the final prediction. This U-shaped network structure enables the model to capture and preserve the underlying characteristics of the image through skip connections, which are often overlooked. In our study, a fragmentation module and an aggregation module are integrated within the MLP of the transformer layer, which is designed to mimic knowledge paradigms inspired by human brain learning patterns, thereby facilitating improved information acquisition and processing. A more comprehensive exposition of these modifications is given in the section of transformers layer and the multi-layer perceptron for an easy understanding of our approach.

### Transformers layer and multi-layer perceptron

The transformer layer, a crucial component of the network architecture, consists of the MSA module and the MLP module. The MSA module operates on the entire embedded sequence, extracting potential features, generating valuable features, and eliminating irrelevant noises. It focuses on the most important parts to enhance the quality of the extracted features. The resulting features are then passed to the MLP module for further processing. Within the MLP module, there are two linear projections to transform the features and one dropout layer to prevent overfitting. Here, the Focus and LogSparse modules are used as examples to describe the feature map dimension transformations within the transformer layer. Starting with an input vector in the dimension of $\frac{W}{16}\, \times \,\frac{H}{16}\, \times \,4C$, and after a series of data transformations, including layer normalization and MSA, the dimensions remain unchanged. The Focus module divides the feature map into four contiguous blocks, effectively increasing the number of channels while reducing the length and width to half, thus the dimension will become $\frac{W}{32}\, \times \,\frac{H}{32}\, \times \,16C$. On the other hand, the LogSparse attention module calculates the attention using a mechanism similar to the transformer, preserving the tensor's dimensions without altering them. The BottleneckCSP module conducts two consecutive convolutions, keeping the height and width unchanged while quadrupling the number of channels ($\frac{W}{16}\, \times \,\frac{H}{16}\, \times \,16C$). To maintain the consistency, the C3ECA module also quadruples the number of channels. Both ProbSparse attention and LogSparse attention achieve information aggregation through attention computation, keeping the dimensions of the feature map unchanged. The C3CBAM module has the ability to simultaneously control channel and spatial attention. To bring the feature map back to its initial size, one more convolution is conducted at the end of the computation. Finally, the human brain-inspired learning paradigm implemented in the MLP module is illustrated in Fig. 1.

The MLP module incorporates the fragmentation and aggregation modules as shown in Fig. 9. Although the original MLP module has a simple structure, it can be modified to adapt to specific requirements and improve its performance. Since the hidden layer features are extracted early in the MSA module, the feature maps passed into the MLP module contain a large amount of information, which may introduce ambiguity. To address it, the high-dimensional information is first fragmented, which effectively enhances the information learning rate and reduces the computational complexity from $o\left( \cdot \right)$ to $O\,\left( + \right)$ by replacing cumulative multiplication with cumulative addition. The fragmented information retains the essential correlations between features, enabling more efficient information processing. Subsequently, the information aggregation module operates on the fragmented information and leverages the strong correlations between them. It aggregates the information according to its correlation and completes the construction of the entire learning paradigm. Such integration improves information utilization while minimizing the loss of valuable data, which is the superiority of the human learning paradigm. By incorporating the MSA and MLP modules within the transformer layer, the network architecture has the benefits of feature extraction, noise reduction, fragmentation, and information integration, which contribute to the overall performance and effectiveness of the proposed network.

Further explanations for the core illustrations of the technical methods mentioned above are given to clarify their relationships. Figure 1 elucidates the introduced human learning paradigm and outlines its knowledge framework. Figure 8 depicts the framework of the baseline model with our learning paradigm embedded in the model’s transformer layer. The overall module of integrating the paradigm within the MLP section of the transformer layer is shown in Fig. 9. Altogether, these depictions aim to clarify how the human learning paradigm is implemented within the MLP in the transformer layer.

### Fragmentation module

The Focus module periodically extracts pixels from a high-resolution image and then rebuilds them into a low-resolution image by stacking four neighbors to map the width- and height-dimensional information into the c-channel, enhancing each pixel's perceptual field and minimizing the information loss. In short, the Focus module performs fragmentation operations by proportionally dividing the feature map along three dimensions: length, width, and height. The hardswish activation function in Eq. 1 was employed in the original Focus module [49]. In our model, it was replaced by the SiLU activation function in Eq. 2 because the pixel values of breast fibroadenomas in sonography do not require many boundary constraints [50]:

1 $${\text{hardswish}}({\text{x}})=\left\{\begin{array}{cc}0,& {\text{x}}\le -3\\ {\text{x}},& {\text{x}}\ge 3\\ \frac{{\text{x}}({\text{x}}+3)}{6},& {\text{otherwise}}\end{array},\right.$$

2 $${\text{SiLU}}\left({\text{x}}\right)={\text{x}}\cdot {\text{sigmoid}}\left({\text{x}}\right)=\frac{{\text{x}}}{1+{{\text{e}}}^{-{\text{x}}}}.$$

The BottleneckCSP module consists of a bottleneck block and the main module from CSPNet. The feature map is divided into two parts before entering BottleneckCSP in both length and width dimensions. One part is computed by a series of convolution blocks (1 × 1), and the other is directly fused with the original features by a shortcut. Finally, the fused feature map will be resized to the initial channel dimension using a 1 × 1 convolution block. This module efficiently reduces memory usage and computational bottlenecks because of its lightweight design and strong feature extraction capabilities. The activation functions used in the BottleneckCSP for object detection are Hardswish and LeakyRelu:

3 $$LeakyRelu\left( x \right)\, = \,\left\{ \begin{gathered} 0.01x,\quad x < \,\,0 \hfill \\ x,\quad x\,\, \ge \,0 \hfill \\ \end{gathered} \right.$$

Since the boundary delineation of the activation function is less required in sonography segmentation, we replaced the activation function with SiLU.

The C3ECA module consists of the C3 and ECABottleneck modules and the concept of the slice, processing each block by slicing the pixels of each channel into a defined size. Upon entering the C3ECA module, the feature map undergoes a sequence of three consecutive convolution blocks (1 × 1, 3 × 3, and 1 × 1) for feature slicing. Subsequently, it proceeds through an average pooling layer to capture pertinent information. Finally, a 1 × 1 convolution and an activation function are applied to resize the channel to the initial dimension and ensure feature usability, respectively. To increase the performance without sacrificing the information, a skip connection is added to the C3 module to fuse the feature map before slicing and after processing. The feature fusion can ensure that the feature information after slicing still maintains trustworthiness.

### Aggregation module

**Fig. 10:**
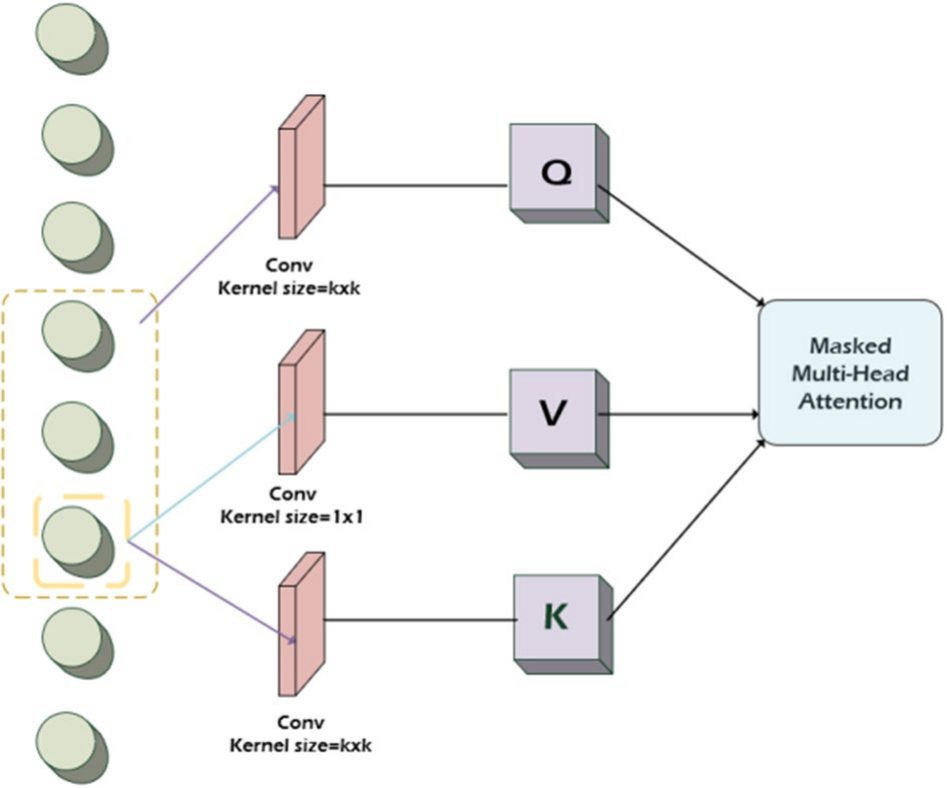
The self-attention convolution mechanism in the LogSparse Attention module

We applied the convolutional self-attention mechanism which is capable of convolutionally transforming input into queries/keys in the network [34]. In comparison to the original transformer design, its location-aware capability can accurately match the most relevant input elements, and LogSparse Attention can read more contextual information to enhance the internal location and sonography perception (see Fig. 10). Most significantly, the network is able to integrate and purify information from each slice for lesion awareness.

The CBAM's spatial and channel attention mechanisms have been shown to improve the network performance in Yolo v5 but with more computational complexity [35]. Therefore, we combined the C3 module, the convolutional block attention module (CBAM), and the Bottleneck module as the C3CBAM Bottleneck module to keep the computation under control. With the integration of spatial and channel attention mechanisms, the meaningful part of the fragmented information is effectively selected, and each piece of information is successfully located to assure its validity.

According to [51], we derived a probabilistic formal approach for convolutional kernel smoothing. Equations 4 and 5 describe the MSA mechanism [13] and the modified attention mechanism, respectively:

4 $$Attention\,\left( {Q,\,K,\,V} \right)\, = \,{\text{softmax}}\,\left( {\frac{{QK^{T} }}{{\sqrt {d_{k} } }}} \right)\,V,$$

5 $$Attention\,\left( {Q,\,K,\,V} \right)\, = \,{\text{softmax}}\,\left( {\frac{{\overline{Q} K^{T} }}{\sqrt d }} \right)\,V,$$

where $\overline{Q}$ is a sparse matrix of the same size as *Q,* and it only contains the Top-u queries under the sparsity measurement *M*(*q*, *K*). It is expected that ProbSparse Attention using the adaptive convolutional kernel approach may perform better in terms of location retention and lengthy sequence prediction, and the addition of the sparse attention coefficients can improve the capture ability of scattered pixels. Information fragmentation may make the image features more confusing, and ProbSparse Attention is able to control the semantic information extraction through smoothly stretched convolutional kernels, which improves the utilization of semantic information and reduces the interference caused by scattered information in the network simultaneously.

### Training and data augmentation

Python 3.7 and PyTorch 1.11.0 were used for the compilation. To decrease the potential for overfitting and regularize the network better, several data augment strategies were applied. With a maximum center and a random offset of 20% from the original image, 128 pixels in each dimension were randomly cropped. Additionally, during data improvement, more images were added to the training dataset by rotating the cropped images with a 20% probability up to $60^{ \circ }$and mirroring them with a 50% probability. Stochastic gradient descent (SGD) was utilized as the optimizer for the training model with an initial learning rate of 0.01, a momentum of 0.9, a weight decay factor of 1e-4, and a default batch size of 24 for 150 epochs. The training was carried out using a single Nvidia Tesla V100 32 GB GPU.

### Evaluation metrics

The similarity between the ground truth and the segmentation is assessed by employing several comparison metrics. Dice similarity coefficient (DSC) was used to compare the areas based on their overlap, and Hausdorff distance **(**HD) was defined as the distance between the boundaries of the ground truth and the segmentation result [52]. The DSC is a widely accepted measure for assessing the overlap between the predicted and ground truth segmentation masks, providing insight into the accuracy of the segmentation process. Additionally, the HD metric quantifies the maximum distance between the contours of the predicted and ground truth regions, offering valuable information about boundary localization accuracy:

6 $$DSC\,\left( {I_{gt} ,\,I_{pt} } \right)\, = \,\,\frac{{2\,\,\left| {I_{gt} \cap I_{pt} } \right|}}{{\left| {I_{gt} } \right| + \left| {I_{pt} } \right|}},$$

7 $$HD\, = \,\left( {\mathop {\max }\limits_{i \in seg} \left( {\mathop {\min }\limits_{j \in gt} \left( {d\left( {i,j} \right)} \right)} \right),\,\mathop {\max }\limits_{j \in gt} \left( {\mathop {\min }\limits_{i \in seg} \left( {d\left( {i,\,j} \right)} \right)} \right)} \right),$$

where *I*_*gt*_ is the ground truth mask, *I*_*pt*_ is the predicted mask, *i* and *j* are points belonging to different sets, and *d* represents the distance between *i* and *j*.

## Data Availability

The datasets generated and/or analyzed during the current study are not publicly available due to security of research data concerns but are available from the corresponding author upon reasonable request.

## Abbreviations

DSC: Dice similarity coefficient
HD: Hausdorff distance
CNNs: Convolution neural networks
BUS: Breast ultrasound
NLP: Natural language processing
C3: Concentrated-comprehensive convolutions
CSPNet: Cross-stage partial network
ECA: Efficient channel attention
CBAM: Convolutional block attention module
DCE-MRI: Dynamic contrast-enhanced magnetic resonance imaging
MSA: Multi-head self-attention mechanism
MLP: Multi-layer perceptron
